# Differentiation of SH-SY5Y neuroblastoma cells using retinoic acid and BDNF: a model for neuronal and synaptic differentiation in neurodegeneration

**DOI:** 10.1007/s11626-024-00948-6

**Published:** 2024-07-17

**Authors:** Imogen L. Targett, Lucy A. Crompton, Myra E. Conway, Tim J. Craig

**Affiliations:** 1 Centre for Research in Biosciences, School of Applied Sciences, University of the West of England, Bristol, BS16 1QY UK; 2 University of Derby, Derby, DE22 1GB UK

**Keywords:** SH-SY5Y, Differentiation, Cholinergic, Synaptic markers, Neurodegeneration

## Abstract

There has been much interest in the use of cell culture models of neurones, to avoid the animal welfare and cost issues of using primary and human-induced pluripotent stem cell (hiPSC)-derived neurones respectively. The human neuroblastoma cell line, SH-SY5Y, is extensively used in laboratories as they can be readily expanded, are of low cost and can be differentiated into neuronal-like cells. However, much debate remains as to their phenotype once differentiated, and their ability to recapitulate the physiology of *bona fide* neurones. Here, we characterise a differentiation protocol using retinoic acid and BDNF, which results in extensive neurite outgrowth/branching within 10 days, and expression of key neuronal and synaptic markers. We propose that these differentiated SH-SY5Y cells may be a useful substitute for primary or hiPSC-derived neurones for cell biology studies, in order to reduce costs and animal usage. We further propose that this characterised differentiation timecourse could be used as an in vitro model for neuronal differentiation, for proof-of principle studies on neurogenesis, e.g. relating to neurodegenerative diseases. Finally, we demonstrate profound changes in Tau phosphorylation during differentiation of these cells, suggesting that they should not be used for neurodegeneration studies in their undifferentiated state.

## Introduction

Reliable cell culture models of neurones are notoriously problematic. As a general rule, researchers either use primary neurones isolated from rodents (e.g. Sahu *et al*. 2019) or, more recently, human-induced pluripotent stem cell-derived (hIPSC) neurones e.g. (Stathakos *et al*. 2021). Both of these systems have their own merits and disadvantages, however both are extremely costly and therefore off limits to all but the most highly funded laboratories in high-income countries. In addition, these models are labour intensive and not applicable to high-throughput applications. There is therefore considerable interest in a cost-effective, reproducible cell model which can be readily scaled up to increase the capacity for repetition and reproducibility. Additionally, whilst it is certainly true that 2D cell monocultures fail to accurately represent the complex cellular interactions seen in all multicellular organisms, conversely they provide a simplified, precisely defined model in which to study the individual signalling pathways, the dysfunction of which is the basis of most diseases.

Many different studies have utilised the human-derived neuroblastoma cell line, SH-SY5Y as an in vitro model of neurones, e.g. ( Xicoy *et al.* 2017; Peng *et al*. 2021; Bell and Zempel 2022). This cell line is originally derived from the SK-N-SH cell line taken from a metastatic bone biopsy from a 4 year old patient. Under basal tissue culture conditions, these cells can be rapidly expanded and display an epithelial-like morphology. However, several different protocols exist to differentiate these cells into a more neuronal phenotype. These protocols generally involve serum restriction and exposure to retinoic acid (RA) (Cheung *et al*. 2009), with some protocols utilising nerve growth factor (NGF) (Shipley *et al*. 2016) and/or brain-derived neurotrophic factor (BDNF) (De Medeiros *et al*. 2019; Encinas *et al*. 2000). Differentiation of these cells results in extensive neurite outgrowth and branching, coupled with the expression of markers of mature neurones (e.g. NeuN, β-III tubulin) (Hromadkova *et al*. 2020), with different studies reporting that the resulting cells display either a dopaminergic (Niaz *et al*. 2021) or cholinergic (De Medeiros *et al*. 2019) phenotype. This is of great interest to researchers in the field of neurodegeneration, as these dopaminergic and cholinergic neurones display selective vulnerability in Parkinson’s Disease (Poewe *et al*. 2017) and Alzheimer’s Disease (Masters *et al*. 2015) respectively. However, few studies to date have investigated the ability of these differentiated cells to form synapses or characterised the timecourse of their differentiation. Such characterisation is potentially extremely useful due to the observation that neurogenesis in the dentate gyrus is impaired in both animal models and human Alzheimer’s disease (Moreno-Jiménez *et al*. 2019; Zhou *et al*. 2023), as it would allow the use of SH-SY5Y cells as an in vitro model for neuronal differentiation.

In this study, we have used a differentiation protocol based on the work of de Medieros (de Medieros *et al*., 2019), which uses both RA and BDNF. We confirm their findings that this protocol results in a robust phenotype of differentiated, neuronal-like cells, with extensive neurite outgrowth and branching. Further, we show that these cells express key pre- and post-synaptic markers, and markers of cholinergic and glutamatergic, but not dopaminergic neurones. Further, we demonstrate consistent changes in Tau phosphorylation during differentiation. Taken together, these data imply that this differentiation protocol might be a useful cell-line model for studying disruptions of neuronal differentiation/neurogenesis observed in the AD brain.

## Materials and methods

### SH-SY5Y Cell Culture

The human neuroblastoma SH-SY5Y cell line was obtained from American Type Culture Collection (ATCC, Manassas, VA). Cells were cultured in Dulbecco’s modified Eagle’s medium F-12 (DMEM/F-12; Gibco, Grand Island, NY, 11,524,436) with GlutaMAX™ supplement, 10% fetal bovine serum (FBS; Gibco, 11,514,436) and 1% non-essential amino acids (NEAAs; Gibco, 11,140,050). Cells were maintained at 37 °C and 5% CO_2_ in humidified air. Once wells reached 70–80% confluency, the cells were subcultured and used up to passage 20. Cells were regularly checked for mycoplasma contamination (Eurofins), returning negative results on every occasion.

### Differentiation time course

**Fig. 1:**
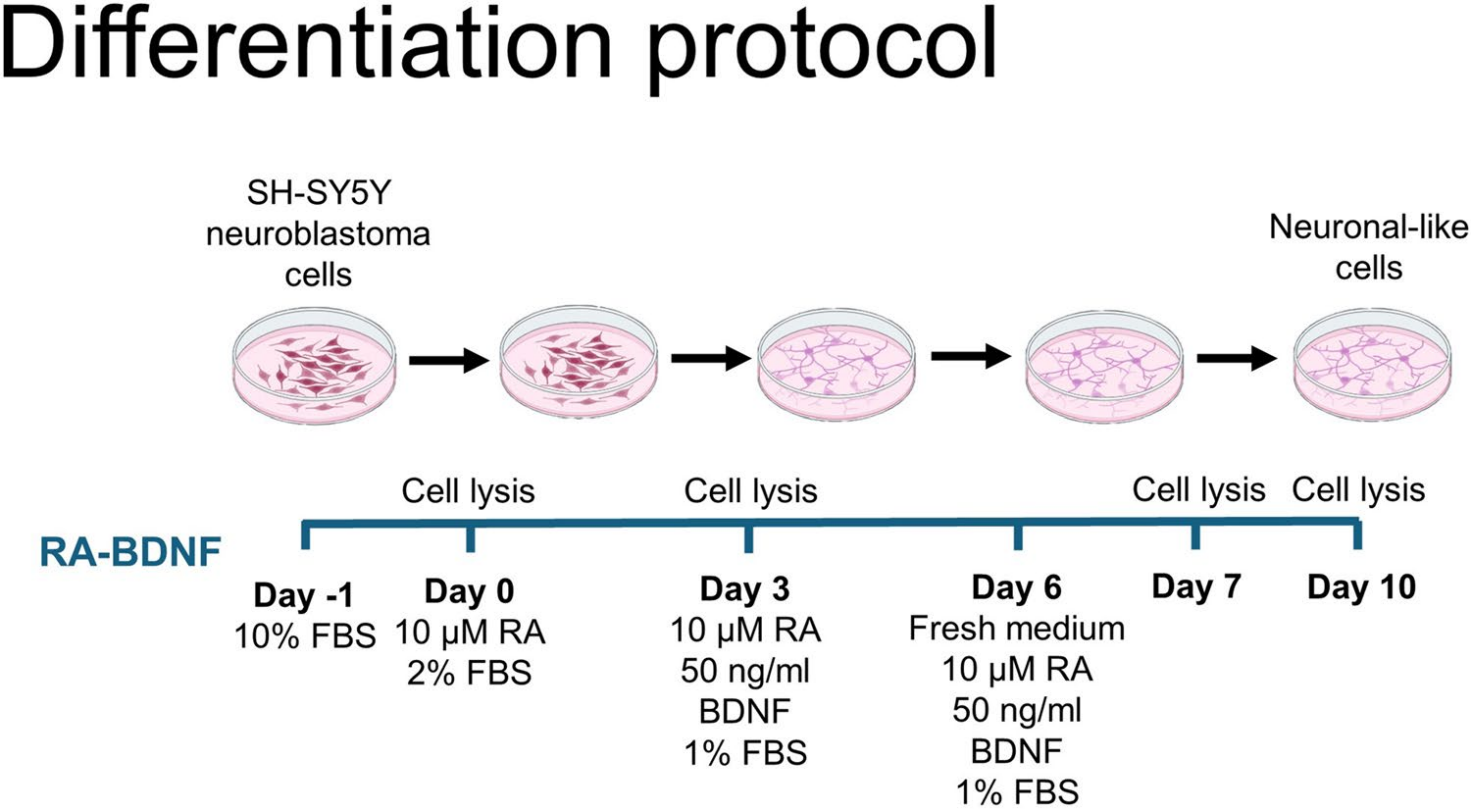
Diagrammatic representation of the differentiation protocol. SH-SY5Y cells are seeded at 40,000 cells per well of a 12-well plate in complete media at D-1. This media is changed to diff1 media at D0 (10 uM RA, 2% FBS), followed by diff2 media at D3 (10 uM RA, 50 ng/ml BDNF, 1% FBS). A further media change into fresh diff2 media is performed at D6. Cells are lysed for timepoints at D0, D3, D7 and D10.

On Day -1, cells were seeded at a density of 10,526 cells/cm^2^ in DMEM/F-12 supplemented with 10% FBS and 1% NEAAs. On Day 0, the cells were washed with 1 × phosphate buffer saline (PBS) and medium was replaced with diff1 media (DMEM/F-12, 2% FBS, 1% NEAAs, 10 μM RA). On Day 3 and 6, cells were washed, and the medium was replaced with diff2 media (DMEM/F-12, 1% FBS, 1% NEAAs, 10 μM RA, 50 ng/ml BDNF). See Fig. 1.

### Cell Lysis + Western blotting

Cells in 12-well plates were lysed on Day 0, 3, 7 and 10 in ice-cold lysis buffer (25 mM Hepes, 150 mM NaCl, 0.1% SDS, 1% triton, phosphatase/protease inhibitor tablet, pH 7.4). Cells were spun by centrifugation (20,000 xg, 4 °C, 5 min) and supernatant stored at -20 °C until required. Protein concentrations were quantified using a Bradford assay and diluted with lysis buffer to equal concentrations. Protein samples were heated at 75 °C for 5 min, separated by 12% sodium dodecyl sulfate polyacrylamide gel electrophoresis (SDS-PAGE) and transferred to methanol-activated polyvinylidene (PVDF) membrane. Membranes were blocked in 5% milk in 1 × tris-buffered saline-tween 20 (TBST; 10 mM Tris–HCl (pH 7.6), 150 mM NaCl, and 0.1% Tween-20) for 1 h at room temperature (RT) and incubated with primary antibodies (Table 1) in 5% milk at 4 °C overnight. Membranes were subsequently washed and incubated with secondary antibodies (Table 2) in 5% milk for 1–2 h in the dark at RT. Membranes were treated with HRP substrate and analysed using LICOR Image Studio software. All protein levels were normalised to GAPDH levels.

**Table 1:** List of primary antibodies used for Western blotting and immunohistochemistry

Primary Antibody	Species	Company	Cat number	Dilution
Post-synaptic density-95 (PSD-95) (D27E11)	Rabbit monoclonal	Cell Signaling technologies	3450	1:500
PSD95-Specific,DLG4	Rabbit polyclonal	Protein Tech	20665–1-AP	1:500
β-III tubulin (D71G9)	Rabbit monoclonal	Cell Signaling technologies	5568	1:2000 WB
Anti-β III tubulin antibody (2G10)	Mouse	Abcam	ab78078	1:700 ICC
Synaptophysin (D8F6H)	Rabbit monoclonal	Cell Signaling technologies	36406	1:20,000 WB, 1:500 ICC
Synaptophysin	Mouse monoclonal	Protein Tech	67864–1-Ig	1:400
Recombinant Anti-Tau (phospho S396)	Rabbit monoclonal	Abcam	ab109390	1:2000
Recombinant Anti-Tau (phospho S202 + T205)	Rabbit monoclonal	Abcam	ab210703	1:1000
Tau (D1M9X)	Rabbit monoclonal	Cell Signaling technologies	46687	1:1000
Recombinant Anti-Choline Acetyltransferase (ChAT)	Rabbit monoclonal	Abcam	ab181023	1:1000
Tyrosine Hydroxylase (TH)	Rabbit polyclonal	Protein Tech	25859–1-AP	1:1000
Glutamate transporters glutamate-aspartate transporter (GLAST)	Rabbit polyclonal	Protein Tech	20785–1-AP	1:500
Glutamine Synthetase (GLUL)	Mouse monoclonal	Protein Tech	66323–1-Ig	1:1000
Glyceraldehyde 3-phosphate dehydrogenase (GAPDH)	Mouse monoclonal	Santa Cruz biotechnologies	sc47724	1:500

**Table 2:** List of secondary antibodies used for Western blotting and immunohistochemistry

Secondary Antibody	Company	Cat number	Dilution
**Western Blotting**			
Goat anti-Rabbit IgG (H + L) Secondary Antibody, HRP	Invitrogen	15217664	1:10,000
Goat anti-Mouse IgG (H + L) Cross-Adsorbed Secondary Antibody	Invitrogen	15291378	1:10,000
IRDye 800CW Donkey anti-Mouse	Li-cor Biosciences	925–32212	1:20,000
**Immunocytochemistry**			
Goat anti-Rabbit IgG (H + L) Highly Cross-Adsorbed Secondary Antibody, Alexa Fluor™ 488	Invitrogen	10236882	1:1000
Invitrogen Goat anti-Mouse IgG (H + L) Highly Cross-Adsorbed Secondary Antibody, Alexa Fluor™ 568	Invitrogen	10226882	1:1000

### Immunocytochemistry

Cells were seeded on Geltrex-coated coverslips in 24-well plates. 10 × Geltrex was thawed with ice-cold serum free (SF) DMEM/F12 media and diluted to 0.25 × Geltrex with SF DMEM/F-12. 80 µL of 0.25 × Geltrex was added to each coverslip (1.3 cm^2^) and incubated for at least 20 min at 37 °C. Geltrex was removed, and cells (10,526 cells/cm^2^) were added to each coverslip and incubated for 4 h before each well was flooded with 200 µL DMEM/F-12 (10% FBS, 1% NEAAs).

After the differentiation protocol was followed, cells were fixed with 4% paraformaldehyde for 15 min at RT. Cells were washed with 1 × PBS three times and stored overnight at 4 °C. Blocking/permeabilisation was carried out in 10% horse serum, 1% BSA, in 0.1% PBTx (1 × PBS + 0.1% Triton-X) for 1 h. Primary antibodies β-III tubulin, PSD-95 and synaptophysin (Table 1) were incubated in 1% horse serum, 0.1% BSA in 0.1% PBTx overnight at 4 °C in a humid chamber with 1 × PBS. Coverslips were washed three times with 1 × PBS and incubated with secondary fluorescent antibodies (Table 2; Alexa Fluor 488 (AF488) and AF568 at a 1:1000 dilution) for 1–2 h at RT. Cells were washed and cell nuclei were co-stained using a DAPI solution for 5 min. Cells were washed again, and coverslips were mounted onto slides in MOWIOL (25 mg/ml DABICO) media. Cells were visualised and the fluorescent intensities were analysed using a confocal microscope and ImageJ software.

### Statistical analysis

Quantitative data are expressed as mean ± standard error of the mean (SEM) from three independent experiments. Statistical analyses were performed using Prism 9.5.1 and *p*-value was determined as ns, * *p* > 0.05, ** *p* < 0.01, *** *p* < 0.001, **** *p* < 0.0001. Precise statistical tests used will be indicated in individual results.

## Results and discussion

Several papers document a variety of differentiation protocols for SH-SY5Y cells, and these protocols have been characterised to differing extents. As our work is principally focussed on neurodegenerative diseases, we were interested in protocols reporting to induce cholinergic or dopaminergic neuronal phenotypes, as these are relevant to Alzheimer’s Disease and Parkinson’s Disease, respectively. For this reason, we focussed on a differentiation protocol using both retinoic acid (RA) and brain-derived neurotrophic factor (BDNF), with a 10-day timecourse (De Medeiros *et al*. 2019) (Fig. 1). Importantly, this protocol is easy to perform without complex or expensive media preparations; requires no extra cell passaging (in comparison to some published protocols (Shipley *et al*. 2016)); and results in lysates with sufficient protein concentration for Western blotting-based quantification of major neuronal proteins (see Figs. 3, 5 and 6).

**Fig. 2:**
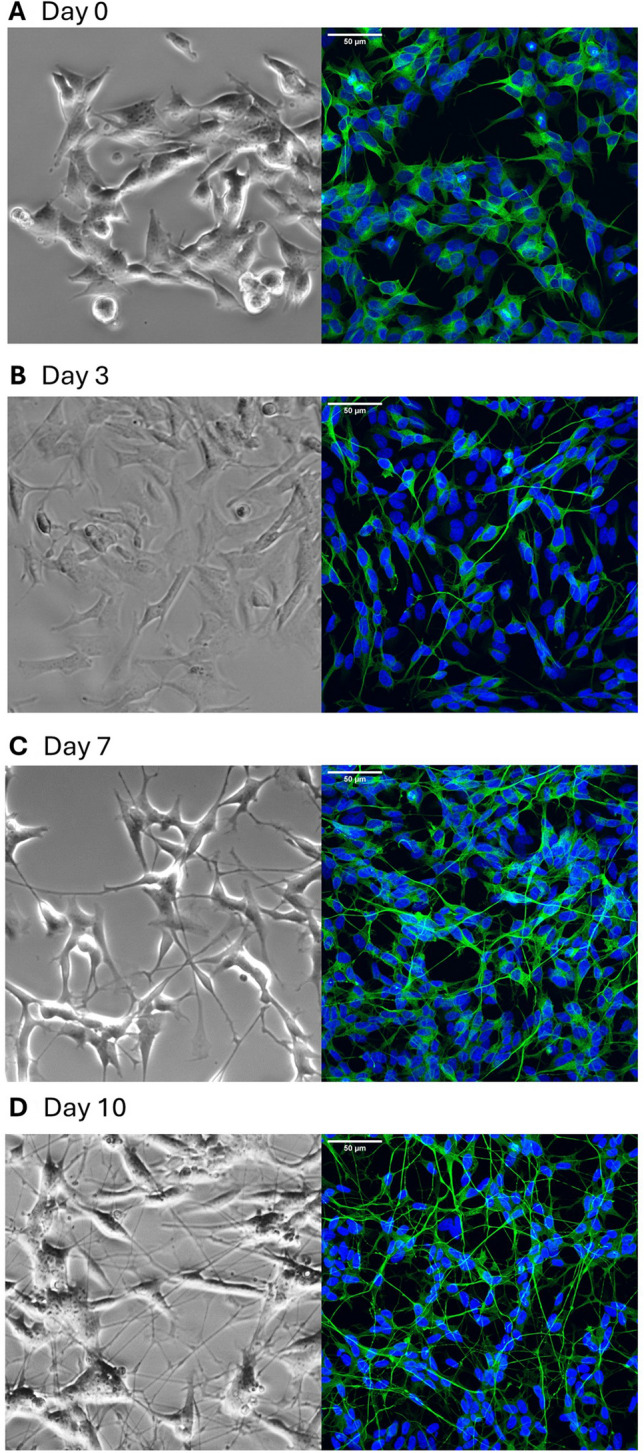
The differentiation protocol leads to reliable and robust outgrowth of neurites expressing β-III tubulin within 10 d. Transmitted light (*left*) and immunocytochemistry (ICC, *right*) images of SH-SY5Y cells at different timepoints during differentiation ((*A*): Day 0, (*B*): Day 3, (***C***): Day 7 and (***D***): Day 10) showing morphological changes during the differentiation. ICC images are stained for β-III tubulin (*green*) and DAPI (*blue*). Images are the composite of 33 confocal slices. Note that the transmitted light and ICC images are taken from different batches of cells. *Scale bar* = 50 µM.

Using this protocol, we observed profound and robust morphological changes over the 10-day timecourse. During this time, the SH-SY5Y cells went from a largely epithelial morphology (Fig. 2*A*) to an increasingly neurone-like morphology with extensive neurite outgrowth and branching by Day 10 (Fig. 2*B,C* and *D*). Additionally, these neurites stained strongly positive for the neurone-specific β-III tubulin (Fig. 2*E–H*). Therefore, the differentiation protocol we have employed results in neuronal morphology in 10 d, driven by β-III tubulin polymerisation along neurites.

**Fig. 3:**
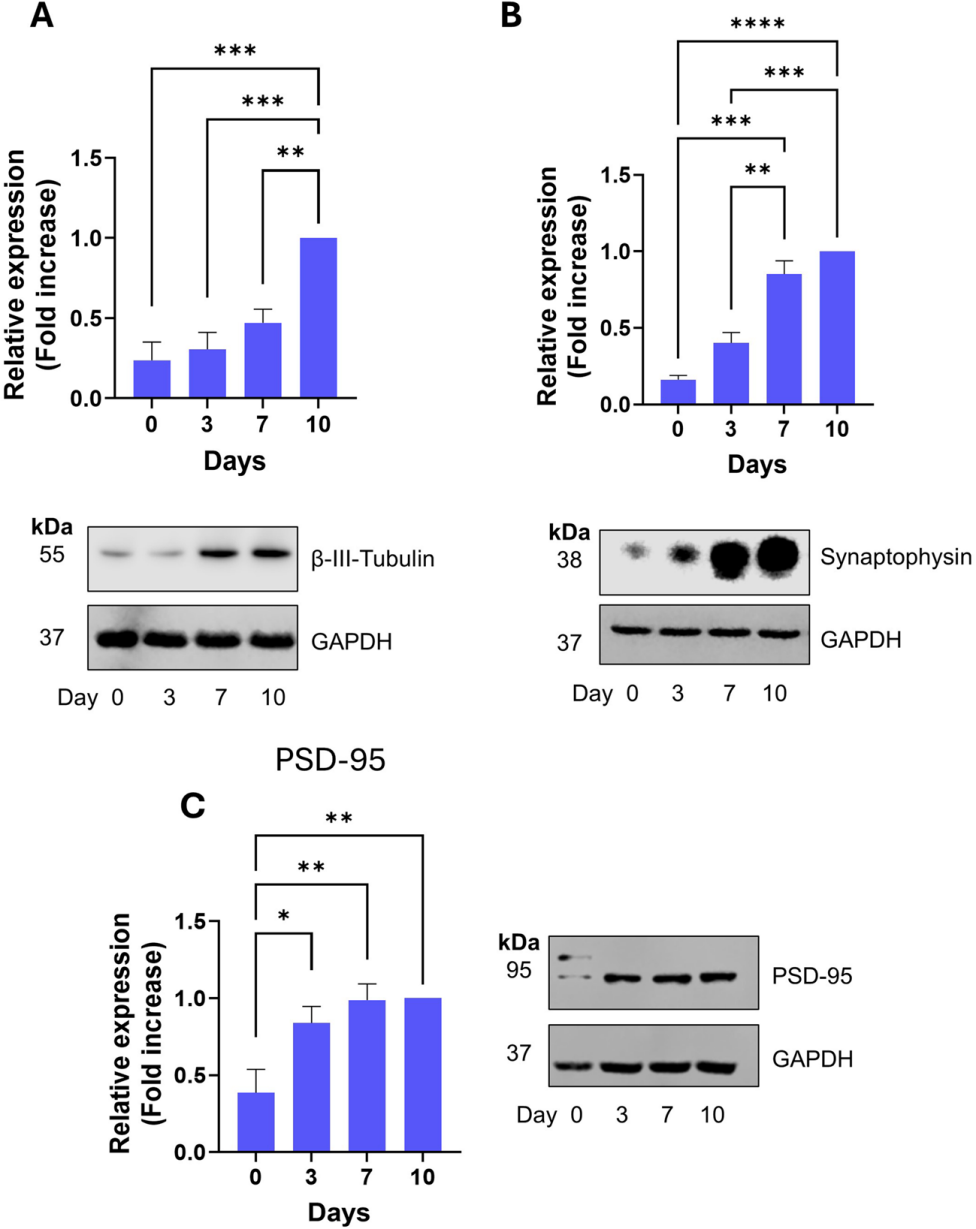
Time-dependent expression of key neuronal and synaptic markers during differentiation of SH-SY5Y cells. All experiments involved SDS-PAGE and Western blotting of whole cell lysates at the designated timepoints. (***A***): Expression of β-III tubulin. *Top*: quantification of the relative expression levels normalised to D10. *Bottom*: representative Western blot. (***B***): Expression of synaptophysin. *Top*: quantification of the relative expression levels normalised to D10. *Bottom*: representative Western blot. (***C***): Expression of PSD-95. *Top*: quantification of the relative expression levels normalised to D10. *Bottom*: representative Western blot. For all *graphs*, values are mean ± SEM (*n* = 3). * *p* < 0.05, ** *p* < 0.01, *** *p* < 0.001, **** *p* < 0.0001. 1-way ANOVA with Tukey’s post-hoc test.

To further characterize this differentiation protocol, we investigated and quantified the expression of key neuronal and synaptic markers during this timecourse using Western blotting (Fig. 3*A-C*). As expected from the ICC results in Fig. 2, we observed a significant and robust induction of β-III tubulin expression across the timecourse of differentiation, reflecting neurite formation. Additionally, we observed a strong induction of the expression of both the pre-synaptic marker, synaptophysin (Fig. 3*B*) and the post-synaptic marker, PSD-95 (Fig. 3*C*). Whilst both markers showed a significant increase over the timecourse of differentiation, it is interesting to note that PSD-95 expression increased most strongly between days 0–3, whereas synaptophysin expression increased most strongly between days 3–7. These results therefore indicate that, as well as neuronal morphology, differentiated SH-SY5Y cells may be forming synaptic structures.

**Fig. 4:**
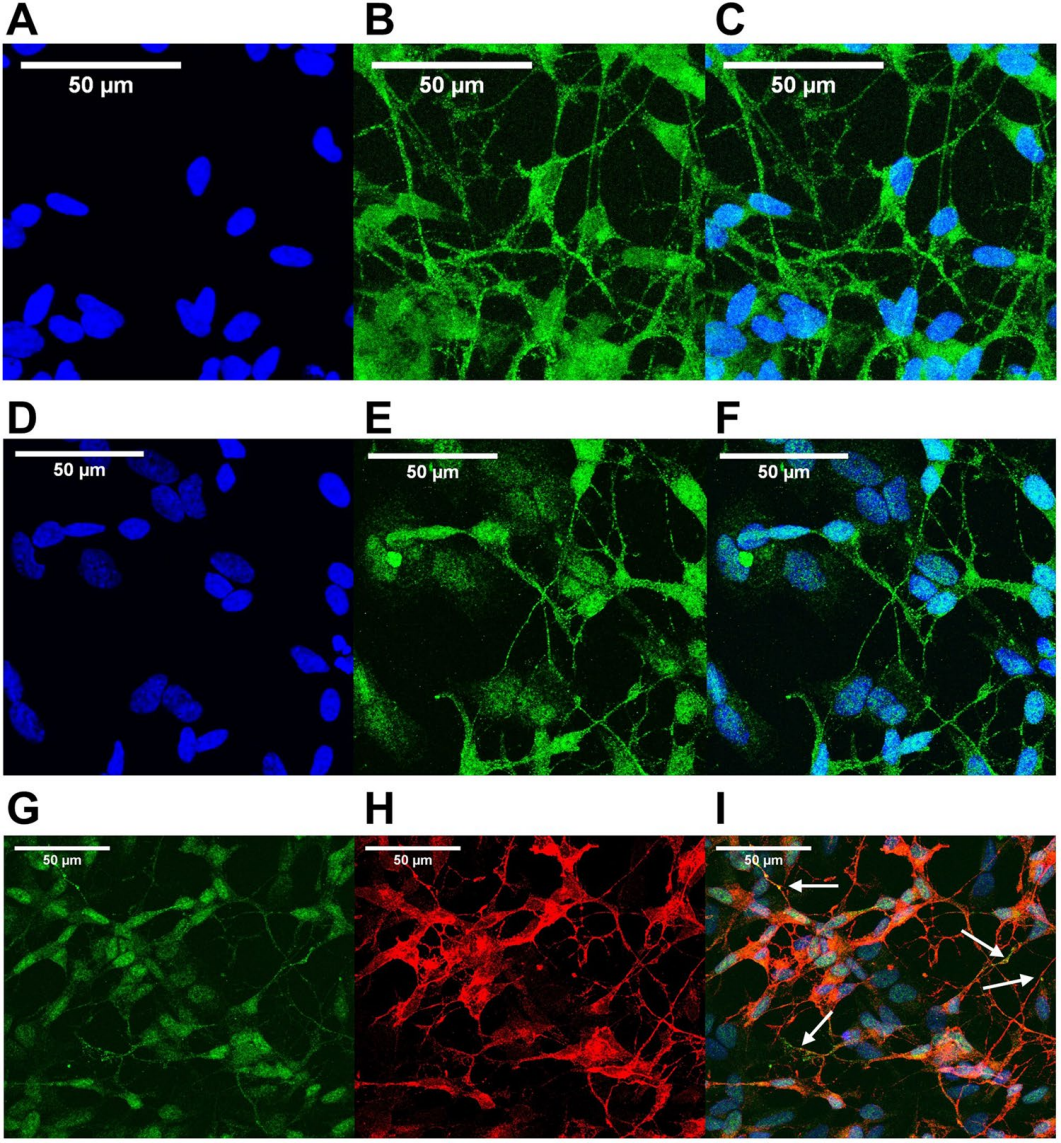
Punctate distribution of pre- and post-synaptic markers along neurites of D10 differentiated SH-SY5Y cells. (**A-C**): Immunocytochemistry for DAPI (*blue*, ***A***), synaptophysin (*green*, **B**) and merged (***C***) in SH-SH5Y cells after 10 d of differentiation, showing diffuse somatic and more punctate neurite localisation. Images are the composite of 33 confocal slices. (***D-F***): Immunocytochemistry for DAPI (*blue*, ***D***), PSD-95 (*green*, ***E***) and merged (***F***) in SH-SH5Y cells after 10 d of differentiation, showing diffuse somatic and more punctate neurite localisation. (***G-I***): Immunocytochemistry for PSD-95 (*green*, ***G***), synaptophysin (*red*, ***H***) and merged (***I***). *Arrows* in ***I*** indicate areas of close apposition of PSD-95 and synaptophysin staining. Images are the composite of 32 confocal slices. *Scale bar* = 50 µM.

Having established that this differentiation protocol results in the expression of synaptic markers, we next used immunocytochemistry to characterise the localisation of these proteins. Our reasoning was that if SH-SY5Y cells differentiated using this protocol are capable of synapse formation, we would observe punctate distribution of both the post-synaptic marker PSD-95 and the pre-synaptic marker synaptophysin. As shown in Fig. 4*A-C*, synaptophysin showed a neurite-localised, punctate distribution by Day 10. This is what we would expect if presynaptic structures were being formed in these cells, and is broadly similar to synaptophysin staining observed in primary neurones, e.g. (Swarnkar *et al*. 2021). We observed a similar distribution of PSD-95 along neurite processes (Fig. 4*D-F*), which was possibly even more punctate than that of synaptophysin in places. Whilst it is notable that both synaptic markers are visible throughout the cell bodies at Day 10, these results nevertheless suggest that this differentiation protocol leads to the formation of both pre- and post-synaptic structures. We note, however, that we should be cautious in over-interpreting these data, and that the puncta we show here may simply represent transport cargo. However, costaining for both PSD-95 and synaptophysin (Fig. 4*G-I*) revealed several sites where these pre- and post-synaptic markers were in close apposition. Whilst we acknowledge that this does not provide definitive evidence, it is however consistent with the formation of synaptic structures.

**Fig. 5:**
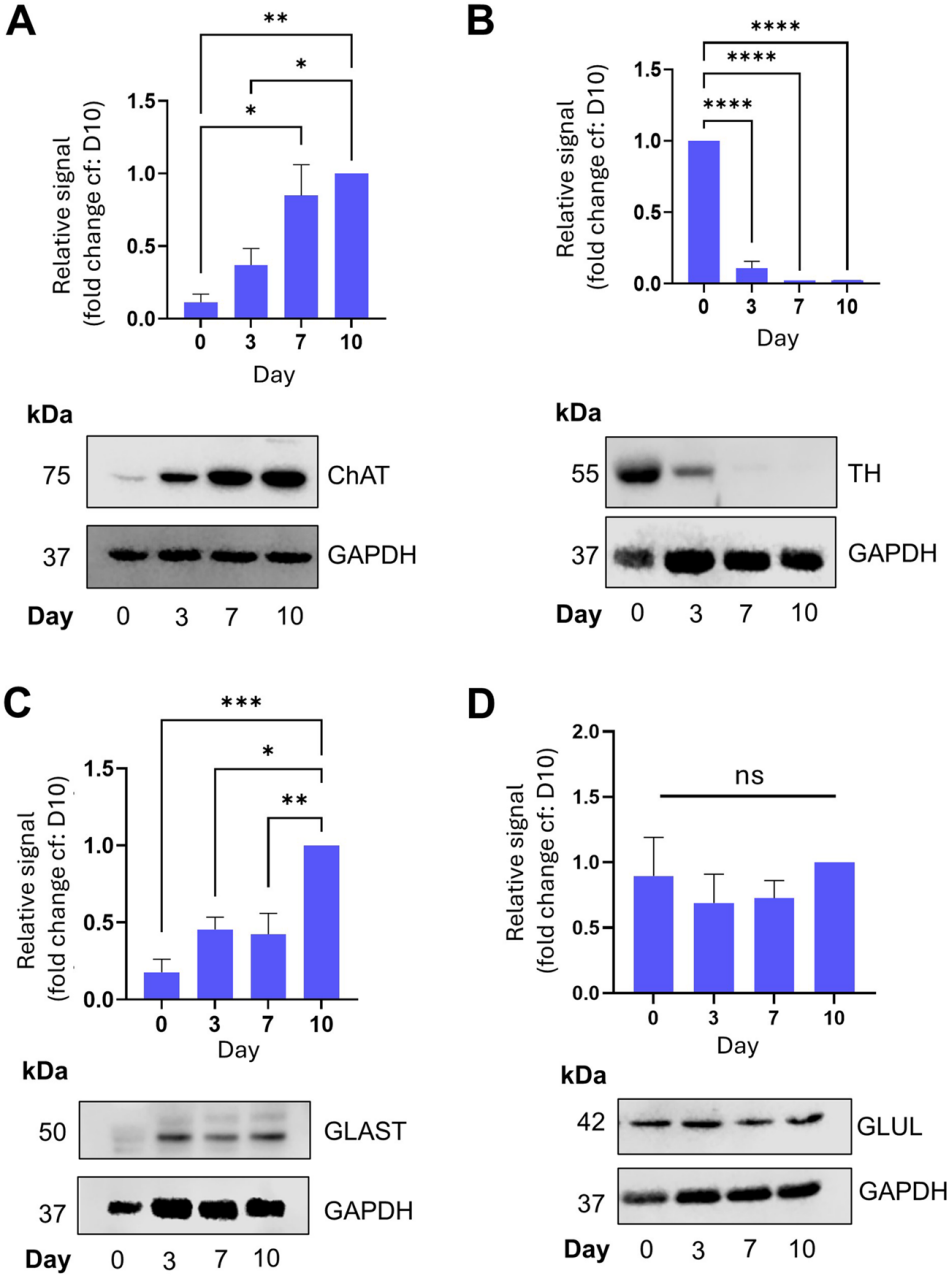
Differentiated cells display a mixed cholinergic/glutamatergic phenotype. All experiments involved SDS-PAGE and Western blotting of whole cell lysates at the designated timepoints. (***A***): Expression of choline acetyltransferase (ChAT). *Top*: quantification of the relative expression levels normalised to D10. *Bottom*: representative Western blot. (***B***): Expression of tyrosine hydroxylase (TH). *Top*: quantification of the relative expression levels normalised to D10. *Bottom*: representative Western blot. (***C***): Expression of glutamate-aspartate transporter (GLAST). *Top*: quantification of the relative expression levels normalised to D10. *Bottom*: representative Western blot. (***D***): Expression of glutamine synthetase (GLUL). *Top*: quantification of the relative expression levels normalised to D10. *Bottom*: representative Western blot. No significant differences observed. For all *graphs*, values are mean ± SEM (*n* = 3). 1-way ANOVA with Tukey’s post-hoc test. * *p* < 0.05, ** *p* < 0.01, *** *p* < 0.001, ****, *p* < 0.0001.

There are differing reports as to the neuronal subtype best represented by differentiated SH-SY5Y cells, with different protocols reporting either a dopaminergic (Niaz *et al*. 2021) or cholinergic phenotype (De Medeiros *et al*. 2019). For this reason, we used Western blotting to examine the presence and relative levels of markers of different neuronal subtypes. Consistent with our differentiated SH-SY5Y cells having a cholinergic phenotype, we observed a robust increase in the expression of choline acetyltransferase (ChAT) over the timecourse of differentiation (Fig. 5*A*). In contrast, expression of tyrosine hydroxylase (TH), a key enzyme in dopamine synthesis, was expressed at high levels on Day 0, however this level rapidly decreased as the cells differentiated, becoming almost undetectable by Day 10 (Fig. 5*B*). Interestingly, expression of glutamate-aspartate transporter (GLAST), a key protein in cellular glutamate uptake and often used as a marker of glutamatergic neurones (Rodríguez-Campuzano and Ortega 2021), was also increased during differentiation (Fig. 5*C*), implying that the fully differentiated cells have either a mixed cholinergic and glutamatergic phenotype or that there is a mixed population. Interestingly, glutamine synthetase (GLUL) levels did not change significantly during our differentiation protocol (Fig. 5*D*), implying that either this is not a reliable marker of glutamatergic neurones, or that our protocol results in an incomplete glutamatergic phenotype. It should be noted that there is scant evidence in the literature of differentiated SH-SY5Y cells expressing AMPA or NMDA receptors, although some studies have demonstrated the expression of mRNA (Anchesi *et al*. 2023) and protein (Yang *et al*. 2020) of both under certain conditions (often a combination of RA and other factors, e.g. GLP-1 or THC).

**Fig. 6:**
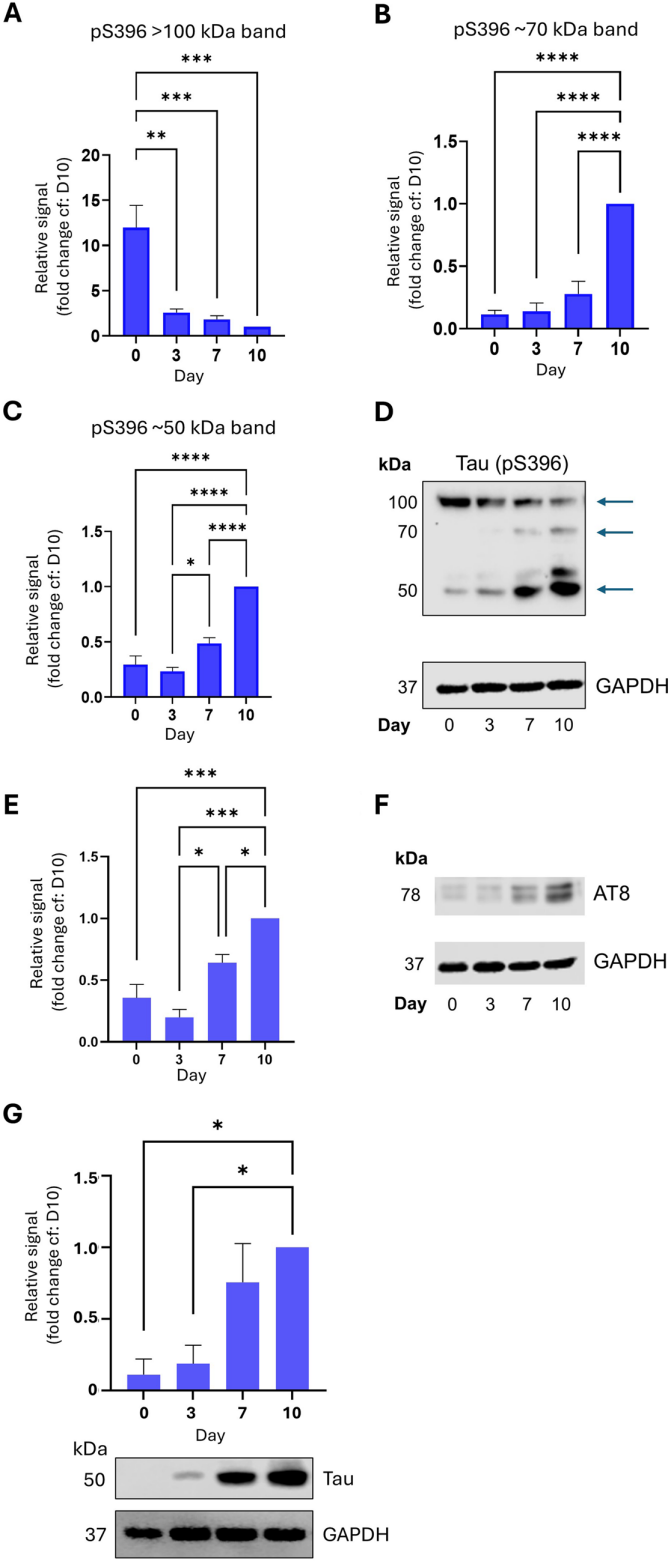
Tau phosphorylation at residues associated with AD pathology displays differentiation-dependent regulation. All experiments involved SDS-PAGE and Western blotting of whole cell lysates at the designated timepoints. (***A-C***): Quantification of 3 separate pS396 bands detected on Western blot. These bands are ~ 100 kDa ((*A*), presumably dimers of phosphorylated Tau (pTau)), ~ 75 kDa ((***B***), presumably either a multiply phosphorylated monomeric pTau or a monomer of a longer isoform also detected by this antibody) and ~ 50 kDa ((***C***), presumably monomeric pTau). Bands quantified are indicated in representative Western blot (***D***). Quantification of the relative signal is normalised to D10. Values are mean ± SEM (*n* = 3). *Bottom*: representative Western blot. (***E***): Quantification of S202/T205 phosphorylation epitope (also referred to as AT8), relative signal levels normalised to D10. (***F***): representative Western blot, with the 78 kDa band quantified indicated with an *arrow*. (***G***): *Above*: Total tau quantification, relative signals normalised to D10. *Below*: Representative Western blot of total tau and GAPDH. For all *graphs*, values are mean ± SEM (*n* = 3). 1-way ANOVA with Tukey’s post-hoc test. * *p* < 0.05, ** *p* < 0.01, *** *p* < 0.001, ****, *p* < 0.0001.

Tau is a microtubule-binding protein which plays important roles in neuronal growth and development including microtubule polymerisation and stabilisation e.g. (Kadavath *et al*. 2015), however, it is best known as the major component of neurofibrillary tangles (NFTs) in Alzheimer’s disease (Masters *et al*. 2015). Indeed several studies have indicated that cognitive decline correlates more closely with the extent of Tau pathology (termed Braak staging) than any other pathological hallmark of Alzheimer’s Disease (Lowe *et al*. 2018). In NFTs, Tau is typically found in a hyperphosphorylated state, leading to the general view that all Tau phosphorylation is neurotoxic. However, this is not always the case, and Tau phosphorylation (even at residues associated with AD-pathology) has been demonstrated to occur during development of neurones and the nervous system (Fuster-Matanzo *et al*. 2009, 2012; Hefti *et al*. 2019). We therefore examined differentiation-dependent regulation of phosphorylation of Tau at three specific residues, S202/T205 (collectively known as the AT8 epitope) and pS396, which are all associated with AD pathology. The pS396 antibody detects bands of 4 different molecular weights – at ~ 50 kDa (presumably representing pS396 monomeric Tau), at > 100 kDa band (presumably oligomers or dimers of pS396 Tau) and two bands between, at approximately 55 and 70 kDa, which we presume represent either multiple phosphorylated forms of Tau or different isoforms. Interestingly, we noticed a reciprocal relationship between the 100 kDa band and the lower molecular weight bands during differentiation, with the 50, 55 and 70 kDa bands increasing and the 100 kDa band decreasing in abundance between Day 0 and Day 10 (Fig. 6*A-D*). These results suggest that during neuronal differentiation, whilst phosphorylation of Tau at pS396 increases, the formation of higher-order structures decreases. In contrast, for the S202/T205 epitope, we noted a consistent increase in Tau phosphorylation across the timecourse. In our hands, this antibody also revealed multiple bands between 75 kDa and > 200 kDa, however it was the doublet at around 78 kDa (the predicted molecular weight according to the manufacturer, see Table 1 for details) which showed a strong developmental trend (Fig. 6*E* and *F*). It should also be noted that total Tau expression is strongly induced by our differentiation protocol (Fig. 6*G*), indicating that this is potentially behind the increase in abundance of phosphorylated species of Tau seen in this study. Importantly, however this increase in total Tau expression is in contrast to the decrease in pS396 Tau oligomers. Whilst we cannot ascribe a specific function to these phosphorylated Tau species in differentiation, the fact that they are differentially developmentally regulated implies that dysregulation of neurogenesis, known to occur in AD, might be an instigator of aberrant Tau phosphorylation. Interestingly, both the S396 and S202/T205 epitopes have been reported to be phosphorylated by CDK5/p35 and GSK3β (Johnson and Stoothoff 2004; Li *et al*. 2006; Gao *et al*. 2020), implying that these kinases are activated during the process of differentiation in SH-SY5Y cells. Indeed, this observation is in line with studies demonstrating a critical role for CDK5 in adult hippocampal neurogenesis (Lagace *et al*. 2008). Thus, the SH-SY5Y differentiation protocol outlined in this study represents a viable and robust model to investigate this.

## Conclusions

In this study, we have provided further evidence that the SH-SY5Y cell line can be reliably differentiated into mature neuronal-like cells, displaying a cholinergic/glutamatergic phenotype, using serum restriction, retinoic acid and BDNF. We have also demonstrated that these differentiated cells exhibit a punctate distribution of key synaptic markers along neurites. Many studies use these cells in their undifferentiated state, but our data argue that this is unwise due to the lack of expression of neuronal proteins. This is in agreement with a recent study showing changes in APP expression and localisation during differentiation in SH-SY5Y cells (Riegerová *et al*. 2021). These data support previous studies (De Medeiros *et al*. 2019; Hromadkova *et al.* 2020) and further suggest that this differentiation protocol is a suitable model for the study of neurogenesis and neuronal differentiation. It is now widely accepted that adult hippocampal neurogenesis occurs in humans, and moreover continues throughout life. Furthermore, this neurogenesis declines with age, and is particularly attenuated in Alzheimer’s disease (Moreno-Jiménez *et al*. 2019). It is noteworthy that one of the first sites affected in AD, the dentate gyrus of the hippocampus, is also one of the main sites of adult neurogenesis (Kempermann *et al*. 2018). Our demonstration that Tau phosphorylation is regulated during differentiation of SH-SY5Y cells implies that dysfunctional differentiation of neuronal precursors in AD may be a contributing factor in aberrant Tau phosphorylation and Tau pathology. We therefore propose that this SH-SY5Y differentiation protocol is a useful model to study this process. Future potential investigations could include studies on the effects of beta-amyloid on this differentiation process, the effect of neuroinflammatory factors, environmental toxins, e.g. ethanol or nanoplastics, dietary factors and the influence of ischaemia, all of which have been suggested to play a role on AD development. Additionally, this system is highly amenable to genetic manipulation to investigate the roles of the many different human polymorphisms associated with AD risk. Whilst we fully acknowledge that our 2D monoculture system does not recapitulate the exquisite complexity of the central nervous system, we nevertheless believe it will prove useful for proof-of-concept investigations or high throughput assays (due to its low cost), which can then be extended into more physiologically relevant systems.

## Data Availability

All raw data is available from the authors on request.
